# New Trends in the Simulation of Nanosplasmonic Optical D-Type Fiber Sensors

**DOI:** 10.3390/s19081772

**Published:** 2019-04-13

**Authors:** Ariel Guerreiro, Diego Felipe Santos, José Manuel Baptista

**Affiliations:** 1 Faculdade de Ciências, Universidade do Porto, Rua do Campo Alegre 687, Porto 4169-007, Portugal; asguerre@fc.up.pt; 2 INESC TEC, Rua do Campo Alegre 687, Porto 4169-007, Portugal; 3 Instituto de Plasmas e Fusão Nuclear, Av. Rovisco Pais 1, Lisboa 1049-001, Portugal; diegonobregasantos@hotmail.com; 4 Faculdade de Ciências Exatas e da Engenharia, Universidade da Madeira, Campus da Penteada, Funchal 9000-390, Portugal

**Keywords:** optical fiber sensors, refractive index sensor, D-type fiber, photonic crystal fiber, metallic nanowires, metamaterials, nanoplasmonics, surface plasmon resonance, optical mode coupling, finite-element method, numerical methods

## Abstract

This article presents a review of the numerical techniques employed in simulating plasmonic optical sensors based on metal-dielectric nanostructures, including examples, ranging from conventional D-type fiber sensors, to those based on photonic crystal D-type fibers and incorporating metamaterials, nanowires, among other new materials and components, results and applications. We start from the fundamental physical processes, such as optical and plasmonic mode coupling, and discuss the implementation of the numerical model, optical response customization and their impact in sensor performance. Finally, we examine future perspectives.

## 1. Introduction

Nowadays, fiber optical sensors integrate information and monitoring systems, as well as different types of devices, with applications from infrastructure and industrial monitoring to environmental control, largely due to their high sensitivity and immunity to electromagnetic and radiofrequency interference [1]. A considerable part of these sensors are refractive index sensors that have been functionalized to respond indirectly to a multitude of parameters, from pH, temperature, humidity, to carbon dioxide (CO_2_) concentration and many other physical, chemical and biological parameters. This technology has benefited from the exploration of surface plasmon resonance (SPR) that occurs at the interface between metal and dielectric films deposited on the surface of optical fibers, reaching sensitivities in excess of 10^−7^ RIU [2].

Still, the recent development of sophisticated fabrication techniques capable of producing optical fibers, incorporating structures that combine dielectric, metallic and semi-conducting materials, with sizes below the micron, reaching in some cases even a few tens of nanometers, has paved the way to consider the development of optical fiber sensors that explore nanophotonic phenomena as sensing principles. In optical structures with these sizes, light can no longer be described by ordinary optical rays or even planewaves, as the strongly localized boundary conditions, imposed at the interface between different materials, force light into optical modes that are strongly dependent of the geometry and material optical properties. This dependency can be explored to customize the optical response and optimize the performance of the sensors, however, a development approach based on their actual construction and testing is very expensive and time consuming. Instead, using numerical models, to simulate the sensor response, constitutes an important design tool to obtain a first and expedite approximation of the performance of a sensor design, that can provide insight on the optical phenomena involved, as well as, a significant reduction of the development and optimization costs.

Typically, the propagation of light in optical structures much larger than the wavelength can be described using ray-tracing models [3,4], whereas the performance of interferometric sensors or those based on overlapping of thin metal-dielectric films usually is calculated using simulations based on the propagation of plane waves, such as the expansion and propagation method (MEP) [5], the method for multilayer structure transfer matrix modeling [6,7], the optical fiber multilayer cylindrical structure [8], the rigorous coupled wave analysis (RCWA) [9] and sometimes wave-mode coupling [10,11]. However, when considering optical structures with sizes of the order or even below the wavelength, and with complex geometries, these methods fail short. Optical devices and nanostructured materials such as photonic crystal fibers (PCF) [12], metamaterials [13], metallic nanowires [14,15,16], etc. require a distinct approach that considers the strongly localized optical modes supported and their mutual influence, that can adequately calculate the spatial distribution of optical power and the phase effects [17], using typically meshing methods. These methods involve the sampling of the electromagnetic field at a specific number of nodes and establishing a relation between them.

One such approach is the finite-difference time domain (FDTD) method [17]. It consists of solving partial differential equations (PDE) in the time domain, being easier to understand and it is a more commonly used method to solve basic problems. Although FDTD provides a good description of the propagation of light in optical structures, it requires a thin regular mesh and consumes large amount of computer memory [18].

Alternatively, the finite element method (FEM) is preferable for waveguiding structures [19,20], such as those characteristic of optical fiber sensors, as it allows to determine the optical modes (sometimes referred as spatial or normal modes) with a relatively low computer memory requirements by considering an irregular mesh that accommodates the specific geometry of the device and their structural components. The FEM is usually used when it is necessary to vary the resolution by which the field is described over different regions of the simulation box. The drawback in using the FEM is that the process to reduce the PDEs for the electromagnetic field into an eigenvalue problem is typically more complex than using FDTD. However, combined with the facility of automated meshing provided by simulation platforms, such as COMSOL or ANSYS, the FEM is becoming the preferred simulation method.

This article reviews some of the theoretical and numerical foundations and the numerical techniques employed in simulating plasmonic optical fiber sensors based on metal-dielectric nanostructures. We describe from conventional multilayer sensors based on D-type fibers, to those based on photonic crystal fibers and incorporating metamaterials, nanowires, among other new materials and components. 

The article is organized as follows: after the introduction and in the second section we review some of the fundamental physical principles that underline the operation of this type of sensors, such as the equations for optical and plasmonic modes and their coupling. In Section 3 we present some of the numerical models used to describe the sensors and how to compute their optical response and sensor performance. In particular, we focus on FEM. Section 4 provides some examples of application of these methods. Finally, Section 5 discusses future perspectives and delivers some concluding remarks.

## 2. The Fundamentals of Nanoplasmonics

The propagation of light in a nonmagnetic optical medium is governed by the Maxwell equations:

(1) $$\nabla \times E\left(r,t\right)=-\partial_{t}B\left(r,t\right)\;\nabla \times B\left(r,t\right)=\mu_{0}\partial_{t}D\left(r,t\right),$$

where E(r,t) is the electric, D(r,t) is the electric displacement, B(r,t) is the magnetic induction and $\mu_{0}$ is the magnetic vacuum permeability. Combining these two equations, using continuous harmonic waves of the form E(r,t)=E(r,ω)exp(iωt) and the constitutive relation $D\left(r,\omega \right)=\epsilon_{0}\;n^{2}\left(r,\omega \right)E\left(r,\omega \right)$, yields the wave equation:

(2) $$\nabla \times \nabla \times E\left(r,\omega \right)-k_{0}^{2}n^{2}\left(r,\omega \right)E\left(r,\omega \right)=0,$$

with r=(x,y,z) and where $\mu_{0}\epsilon_{0}\omega^{2}=k_{0}^{2}$, ω is the angular frequency, $\epsilon_{0}$ is the vacuum permittivity and n(r,ω) is the refractive index, which is a complex quantity and can be decomposed into the real and imaginary parts, say $n\left(r,\omega \right)=n_{r}\left(r,\omega \right)+in_{i}\left(r,\;\omega \right)$. For simplicity, the optical axis is assumed to be the z direction.

This paper considers optical fibers capable of guiding optical and plasmonic excitations characterized by a constant profile of refractive index along the optical axis, i.e., $n\left(r,\omega \right)=n\left(r_{⊥},\omega \right)$, and for which it is possible to apply separation of variable with $E\left(r,\omega \right)=E\left(r_{⊥},\omega \right)\exp\left(-i\beta z\right)$ to obtain:

(3) $$\nabla_{⊥}\times \nabla_{⊥}\times E\left(r_{⊥},\omega \right)-k_{0}^{2}n^{2}\left(r_{⊥},\omega \right)E\left(r_{⊥},\omega \right)+\beta^{2}E\left(r_{⊥},\omega \right)=0.$$

If the optical structures are domains with homogeneous material with well-defined and uniform refractive index n(ω) then, Equation (3) becomes a simpler eigen-problem for each domain m:

(4) $$\nabla_{⊥}\times \nabla_{⊥}\times E\left(r_{⊥},\omega \right)-k_{0}^{2}n_{m}^{2}\left(\omega \right)E\left(r_{⊥},\omega \right)+\beta^{2}E\left(r_{⊥},\omega \right)=0,$$

with a common eigenvalue β, a fixed value $k_{0}$ and subjected to the adequate boundary conditions:

(5) $$\hat{n}\times \left[E_{+}\left(r_{⊥},\omega \right)-E_{-}\left(r_{⊥},\omega \right)\right]=0,\;\hat{n}\times \left[B_{+}\left(r_{⊥},\omega \right)-B_{-}\left(r_{⊥},\omega \right)\right]=0$$

(6) $$\hat{n}.\left[n_{+}\left(r_{⊥},\omega \right)E_{+}\left(r_{⊥},\omega \right)-n_{-}\left(r_{⊥},\omega \right)E_{-}\left(r_{⊥},\omega \right)\right]=\frac{\Sigma }{\epsilon_{0}},\;\hat{n}.\left[B_{+}\left(r_{⊥},\omega \right)-B_{-}\left(r_{⊥},\omega \right)\right]=0$$

where $\hat{n}$ is the unit normal pointing from domain with subscript (+) into that with subscript (−). The boundary conditions state imposes that every component of the magnetic field and the tangential components of the electric field is continuous across the interface while, the normal component of the electric field times has a discontinuity proportional to the induced surface charge density Σ at the boundary. The combination between the eigenvalue problems in each domain and the boundary conditions usually forces the spectrum of eigenvalues to be discrete.

It is common to define the effective refractive index for each mode $n_{eff}$ according to $\beta =n_{eff}k_{0}$, which can also have real and imaginary parts. While the real part of the effective refractive index provides the phase velocity of the guided mode along the fiber, the imaginary part characterizes the losses, typically the physical quantity used to inspect the properties of the environment (such as the refractive index of the analyte). The loss curve can be obtained from the imaginary part of the parameter β or alternatively of the effective refractive index of the mode $n_{eff}\left(\omega \right)$ as $\alpha \left(\omega \right)=2Jm\left(\beta \right)=2Jm\left(n_{eff}\right)k_{0}$.

Multiplying Equation (3) by $E^{*}\left(r_{⊥},\omega \right)$ and integrating along the transverse direction, one obtains:

(7) $$\beta^{2}=\frac{\sum_{m}\int^{\text{​}}E^{*}\left(r_{⊥},\omega \right)\left[k_{0}^{2}\;n_{m}\left(r_{⊥},\omega \right)-\nabla_{⊥}\times \nabla_{⊥}\times \right]E\left(r_{⊥},\omega \right)\;d^{2}r_{⊥}}{\int^{\text{​}}E^{*}\left(r_{⊥},\omega \right)E\left(r_{⊥},\omega \right)\;d^{2}r_{⊥}},$$

which implies that the effective refractive index of each mode is in some sense the average of the refractive index over the domains of the optical structure. The contribution of each domain to $n_{eff}$ is proportional to the relative optical power of the mode in that domain, and therefore for some modes, it is possible to consider only the refractive index structure where the mode is localized. This simplification is convenient to obtain analytical (and sometimes numerical) approximations of the guided modes. For example, the core of an optical fiber is usually a cylinder of radius R of a transparent dielectric with larger refractive index than the surroundings. If their refractive indexes can be assumed to have only a real part, then the guided modes are given in terms of Bessel and von Newmann functions of first kind:

(8) $$E\left(r_{⊥},\omega \right)\;\propto \{\begin{array}{l} J\left(Ur_{⊥}\right)\;for\;0<r_{⊥}<R\\ K\left(Wr_{⊥}\right)\;for\;R<r_{⊥} \end{array}$$

where the modal parameters are defined as:

(9) $$U^{2}=\frac{\int_{0}^{R}E^{*}\left(r_{⊥},\omega \right)\left[\nabla_{⊥}\times \nabla_{⊥}\times \right]E\left(r_{⊥},\omega \right)d^{2}r_{⊥}}{\int^{\text{​}}E^{*}\left(r_{⊥},\omega \right)E\left(r_{⊥},\omega \right)\;d^{2}r_{⊥}}$$

(10) $$W^{2}=\frac{\int_{R}^{\infty }E^{*}\left(r_{⊥},\omega \right)\left[\nabla_{⊥}\times \nabla_{⊥}\times \right]E\left(r_{⊥},\omega \right)\;d^{2}r_{⊥}}{\int^{\text{​}}E^{*}\left(r_{⊥},\omega \right)E\left(r_{⊥},\omega \right)\;d^{2}r_{⊥}}.$$

It results that U is real, while W is imaginary, and the mode is strongly localized in the region $r_{⊥}<R$ with an evanescent part in the cladding. Replacing the core with a metallic wire supporting plasmonic surface waves and if the refractive index in the metal is approximately imaginary, then the mode is evanescent in both metal and cladding:

(11) $$\left(r_{⊥},\omega \right)\;\propto \{\begin{array}{l} I\left(Vr_{⊥}\right)\;for\;0<r_{⊥}<R\\ K\left(Wr_{⊥}\right)\;for\;R<r_{⊥} \end{array},$$

where I is the modified Bessel function. 

Complex optical structures can usually support several localized modes that partially overlap, resulting in the coupling between them. For example, consider the localized modes calculated for a core near a metallic nanowire (or any other two structures capable of supporting localized modes):

(12) $$\nabla_{⊥}\times \nabla_{⊥}\times E_{1}\left(r_{⊥},\omega \right)-k_{0}^{2}n_{1}^{2}\left(r_{⊥},\omega \right)E_{1}\left(r_{⊥},\omega \right)+\beta_{1}^{2}E_{1}\left(r_{⊥},\omega \right)=0$$

(13) $$\nabla_{⊥}\times \nabla_{⊥}\times E_{2}\left(r_{⊥},\omega \right)-k_{0}^{2}n_{2}^{2}\left(r_{⊥},\omega \right)E_{2}\left(r_{⊥},\omega \right)+\beta_{2}^{2}E_{2}\left(r_{⊥},\omega \right)=0.$$

where $n_{1}\left(r_{⊥},\omega \right)$ describes the refractive index structure of the core neglecting the presence of the wires and $n_{2}\left(r_{⊥},\omega \right)$ describes the refractive index structure of the wire neglecting the presence of the core. Notice that the total refractive index distribution combining both the core and the wire satisfies:

(14) $$\left(r_{⊥},\omega \right)=n_{1}\left(r_{⊥},\omega \right)+\Delta n_{1}\left(r_{⊥},\omega \right)=n_{2}\left(r_{⊥},\omega \right)+\Delta n_{2}\left(r_{⊥},\omega \right),$$

where $\Delta n_{1}\left(r_{⊥},\omega \right)$ and $\Delta n_{2}\left(r_{⊥},\omega \right)$ are corrections to the refractive index distributions of $n_{1}\left(r_{⊥},\omega \right)$ and $n_{2}\left(r_{⊥},\omega \right)$ due to the presence of the core and the wire, respectively. Then, replacing in Equation (3) a tentative solution as a linear composition of the modes supported by each of the two structures, say $E\left(r_{⊥},\omega \right)=c_{1}E_{1}\left(r_{⊥},\omega \right)+c_{2}E_{2}\left(r_{⊥},\omega \right)$, one obtains:

(15) $$c_{1}\left[\beta^{2}-\beta_{1}^{2}-k_{0}^{2}\Delta n_{1}\left(r_{⊥},\omega \right)\right]E_{1}\left(r_{⊥},\omega \right)+c_{2}\left[\beta^{2}-\beta_{2}^{2}-k_{0}^{2}\Delta n_{2}\left(r_{⊥},\omega \right)\right]E_{2}\left(r_{⊥},\omega \right)=0.$$

For simplicity, we have assumed that the two localized modes are orthogonal and normalized, in other words that they satisfy $\int^{\text{​}}E_{i}^{*}\left(r_{⊥},\omega \right)E_{j}\left(r_{⊥},\omega \right)\;d^{2}r_{⊥}=\delta_{ij}$, which is not always exactly the case, but constitutes a good approximation for the majority of situations. Then, defining the following integrals:

(16) $$g_{ijk}=g_{ijk}\left(\omega \right)=\int^{\text{​}}E_{i}^{*}\left(r_{⊥},\omega \right)\left[\Delta n_{j}\left(r_{⊥},\omega \right)\right]E_{k}\left(r_{⊥},\omega \right)\;d^{2}r_{⊥},$$

multiplying the previous equation either by $E_{1}^{*}\left(r_{⊥},\omega \right)$ or by $E_{2}^{*}\left(r_{⊥},\omega \right)$ and integrating over the transverse direction results in the following eigen-problem:

(17) $$\left(\left[\begin{array}{cc} \beta_{1}^{2}+k_{0}^{2}g_{111} & k_{0}^{2}g_{122}\\ k_{0}^{2}g_{211} & \beta_{2}^{2}+k_{0}^{2}g_{222} \end{array}\right]-\beta^{2}\right)\;\left[\begin{array}{c} c_{1}\\ c_{2} \end{array}\right]=0,$$

with eigenvalues are $\beta_{\pm }^{2}={\bar{\beta }}^{2}\pm \sqrt{\Delta \beta^{4}+k_{0}^{4}g_{211}g_{122}}$ and eigenvectors:

(18) $${\left[\begin{array}{c} c_{1}\\ c_{2} \end{array}\right]}_{+}=\left[\begin{array}{c} \cos\left(\phi_{+}\right)\\ \sin\left(\phi_{+}\right) \end{array}\right],\;{\left[\begin{array}{c} c_{1}\\ c_{2} \end{array}\right]}_{-}=\left[\begin{array}{c} \cos\left(\phi_{-}\right)\\ \sin\left(\phi_{-}\right) \end{array}\right],$$

with $\bar{\beta }=\sqrt{\left[\beta_{1}^{2}+\beta_{2}^{2}+k_{0}^{2}\left(g_{111}+g_{222}\right)\right]/2}$. $\Delta \beta =\sqrt{\left[\beta_{1}^{2}-\beta_{2}^{2}+k_{0}^{2}\left(g_{111}+g_{222}\right)\right]/2}$ and $\phi_{\pm }=\arctan[\left(k_{0}^{2}g_{122}-\beta_{\pm }^{2}\right)/k_{0}^{2}g_{122}]$. The eigenvectors constitute the modes of the structure that combines both a core and a nanowire, and result from the hybridization of the localized modes supported by both substructures. Obviously, this analysis is quite general and can be applied to any type of substructures beside a core and a nanowire, but is an excellent model to explore the effects of mode hybridization. For example, if the overlap integrals $g_{ijk}$ are small, then $\beta_{+}\approx \beta_{2}$ and $\beta_{-}\approx \beta_{2}$ and the two modes do not couple with each other, unless $\beta_{1}\approx \beta_{2}\approx \beta$. In other words, when the two modes are degenerated in β. In this latter case, corresponding to a crossing point in the dispersion curve of the two localized modes, the coupling and exchange energy between the two localized modes is extremely strong even if the overlap is small. Then, the resonant coupling between the two modes introduces a gap of $\sqrt{2\left[k_{0}^{2}\left(g_{111}+g_{222}\right)\right]}$ in the values of β since:

(19) $$\beta_{\pm }=\beta +\frac{k_{0}^{2}\left(g_{111}+g_{222}\right)}{2\beta }\pm \sqrt{\frac{k_{0}^{2}\left(g_{111}+g_{222}\right)}{2},}$$

In some sense, this resonant coupling resembles the hybridization of atomic orbitals that occurs during the formation of a chemical bond, resulting in the formation of a bonding molecular orbital with a decrease of energy and an anti-bonding molecular orbital with an increase of energy relative to the energy of the original orbitals. The processes of mode hybridization are extremely important for optical sensing as they generate coupling resonances at very specific wavelengths, when the dispersion curves of different localized modes cross and become degenerated. If one of the modes is plasmonic in nature and characterized by strong losses, the coupling at that specific wavelength allows to transfer power from the optical into the plasmonic mode, which is then dissipated, resulting in a peak in the loss curve of the optical mode. The key for optical sensing using this type of processes is to promote the dependency of the properties (usually the dispersion curve) of one of the modes on a specific parameter of an analyte (typically, the refractive index). As that property of the analyte changes, it alters the dispersion curve of the mode and shifts the wavelength of the crossing point and thus the wavelength of the peak in the loss curve.

This simple example of a two-mode hybridization clarifies at a fundamental level the optical processes that underline most sensors based on localized optical and plasmonic modes in micro and nanostructured optical fibers. However, these principles are not limited to just two modes hybridization but can be extended to multiple modes hybridization that, in principle, result in much more critical resonance conditions and increased sensor sensitivities. The following section will elaborate on the implementation of numerical methods targeted at capturing and evaluating these processes, as well as engineering them to improve sensor performance. 

## 3. Simulation Models

As discussed in the previous section, the key for the analysis of the performance of optical fiber sensors with micro- and nanoscale metal-dielectric structures, capable of supporting optical and plasmonic modes, is the analysis of the eigenproblem associated with Equation (3). If the structure of the sensor is composed only by cylindrical metal-dielectric components, such as optical waveguides (cores) and nanowires, for which there are good approximate analytical solutions, it is possible to expedite the calculation of the mode hybridization using simple numerical models [21,22]. Other methods, such as the expansion and propagation method [5] and the rigorous coupled wave analysis [9,23,24] can provide improved results to the mode hybridization. On the other hand, when considering devices composed of multiple layers of different materials, there are specific methods capable of addressing these problems, which include the method for multilayer structure transfer matrix modeling [6,25,26,27,28,29] and the optical fiber multilayer cylindrical structure [8]. Yet these methods have some difficulties for devices with nanostructured irregularities, or more complex and intricated structures. In these cases, one can resort to meshing based methods, such finite difference time domain (FDTD) [30,31,32], spectral methods and finite element methods (FEM) [33,34,35,36,37,38,39,40]. 

As referred earlier, FDTD consists of solving partial differential equations in the time domain, being easier to understand and it is a more commonly used method to solve basic problems. FDTD samples the electromagnetic field in the simulation domain at points in a regular mesh and approximates the spatial derivatives in Equation (3) using finite differences. For example, the gradient in two dimensions of a function ϕ can be approximated using symmetric first order finite differences as:

(20) $$\phi \approx \left(\frac{\phi_{i+1,j}-\phi_{i-1,j}}{2h},\;\frac{\phi_{i,j+1}-\phi_{i,j-1}}{2h}\right),$$

where h is the spacing between the mesh points and the subscripts i and j refer to the order of the mesh point, along each dimension of the grid. In very complex geometries, it is required substructures with different sizes, being necessary a dense mesh of sampling points, which requires large computing memory and often results in long simulation times.

An alternative are the spectral methods, and particularly the Fourier spectral methods, which consider the decomposition of the solution as a sum over a set of base functions, say:

(21) $$E\left(r_{⊥},\omega \right)=\sum_{n}c_{n}u_{n}\left(r_{⊥},\omega \right),$$

which after substitution in Equation (3) also results in a set of algebraic eigenproblems, that can be solved numerically. In some way, these methods extend on the method of mode hybridization described in the previous section and that corresponds to a simple case with only two base functions. In general, the adopted basis is composed of plane waves to take advantage of highly efficient Fast Fourier Transforms implementations available, which reduce memory consumption and computing time. However, such approach is better suited for problems with cyclic or periodic boundary conditions, such as photonic crystals and metamaterials.

Finally, FEM considers the sampling of the field at the nodes of an irregular mesh based on the construction of sub-elements within the structure to be studied. For each structure, the calculations of the field equations are represented discreetly in a system of algebraic equations and are solved by an eigenvalue method. FEM is usually used when it is necessary to analyze the field with different resolutions in different regions of the simulation domain, and thus provides a compromise between accuracy, resolution and consumption of computer memory resources. The drawback is that the quality of the solutions obtained by the FEM methods is strongly dependent of the characteristics of the mesh, and finding the best mesh for each specific problem is not a trivial problem, even using the current automatic meshing provided by commercial simulation tools such as COMSOL Multiphysics or ANSYS. Often it is necessary to adjust the mesh and repeat the simulations until an adequate mesh can be found. The choice depends on the balance of two factors:

(1) Reduction of the numerical error due to rounding and respective propagation during the calculation, which is obtained for meshes with fewer elements and, consequently reduces the number of calculations in the simulations.
(2) Reduction of methodological error associated with the finite element method, which is achieved using more samples of the field, thus requiring grids with more elements. However, there are drawbacks to choosing meshes with many elements as this increases the complexity of simulations, requiring more computational resources and more time to perform calculations.

The balance between these two sources of error (numerical and methodological) is achieved by selecting grids where there is a denser sampling of the field (smaller finite elements) in areas where the field has larger variations and lowering the sampling in areas, where the field is quasi constant or zero. When comparing the different methods available to compute the modes of an optical structure, clearly the FEM method presents the best advantages and will be used in the next section to analyze the performance of several examples of optical fiber sensors.

## 4. Examples of New Nanoplasmonic Optical Fiber Sensors

The previous sections introduced the physical fundaments and compared the main numerical methods behind the modeling of nanostructured optical fiber sensors. In particular, we have shown that the key objective is to determine the optical modes supported by each structure and characterize them in terms of their optical properties, namely the effective refractive index, which allows to determine the losses in each section of the sensors, among other things. Here we have focused on sensors with a uniform refractive index distribution along the entire length of the fiber and whose operation strategy consists in determining the changes in the loss spectrum (i.e., as a function of wavelength) produced by the analyte or changes in the environmental refractive index, however the same principles and methods can be applied to other designs and operation approaches.

Moreover, we have established the importance of the localized modes and how they can couple in determining the features of the loss spectrum of the sensors. In particular, we have shown that the strong hybridization of localized modes when their dispersion curves cross, can introduce important features (such as peaks and deeps) in the loss spectrum that are strongly localized in wavelength.

As a result, one of the key elements of sensor design is to engineer the localized modes not only to promote this hybridization but also, and more importantly, to introduce a strong dependency of some of the localized modes in the parameters that the sensor aims to monitor in order to affect the resonant coupling. The critical nature of the resonant coupling amplifies the impact of the change of external parameters on each localized mode and expresses it, for example, in the loss spectrum of the sensor. The importance of numerical methods in the engendering these sensors results from the facility that it allows in the testing of different designs and the optimization of their performance, as illustrated by the examples depicted in the remaining of this section.

### 4.1. D-Type Fiber Configurations (Conventional and PCF) with Metallic Films 

**Figure 1:**
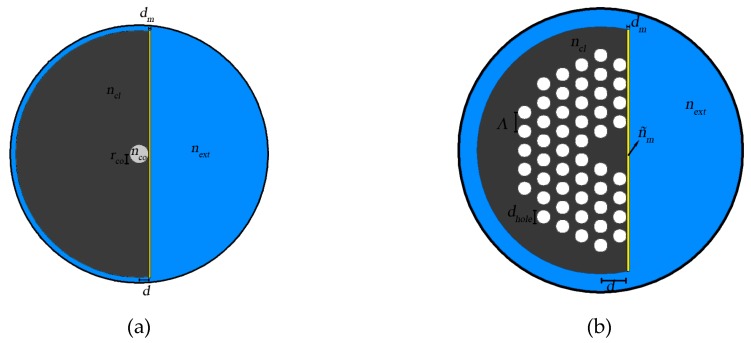
Two SPR D-type fiber configurations: (**a**) conventional and (**b**) PCF. (**a**) Shows the configuration of a conventional D-type optical fiber plasmonic sensor and Figure 1b shows the configuration of PCF D-type optical fiber plasmonic sensor, composed by a core with refractive index (*n_co_*) and surrounded by an array of dielectric structures (the holes in the microstructured fiber) with refractive index equal to the air [41].

Here we compare the impact of engineering a D-type photonic crystal structure in the sensor performance with a more conventional optical structure based on a D-type fiber. In both configurations the flat surface, has been covered with a thin metal layer (Figure 1). The key idea is to explore the role of the photonic crystal nanostructure in customizing the optical properties of the plane wave plasmon modes to ultimately promote enhancement of the sensor performance. 

Using the Sellmeier equation [25] allows one to calculate the refractive index. The fibers have a D-type profile with a metallic layer deposited in the flat surface, whose the refractive index is obtained from the Drude model. It is assumed the analytic medium to be studied, with a refractive index of *n_ext_*, fills the space outside the fiber. The distance between the gold layer and the center of the fiber is *d* - residual cladding, the gold layer thickness is *d_m_*, the holes’ diameter is *d_hole_*, and the holes’ separation is *Λ* – pitch [19].

The conventional D-type fiber is based on a singlemode configuration (*r_co_* = 4 μm), being the edge of the fiber placed at *d* = 4.05 μm from the center of core, the index refraction of the core is *n_co_* = 1.476 and for the cladding is *n_cl_* = 1.452, for a wavelength of 900 nm. In the case of the PCF D-type fiber, the configuration is *d_hole_* = 1.61 μm, *Λ* = 2.3 μm and *d* = 3.2 μm. For both fibers, the metal thickness is *d_m_* 45 nm, and the sensor length is *L* = 1 mm.

**Figure 2:**
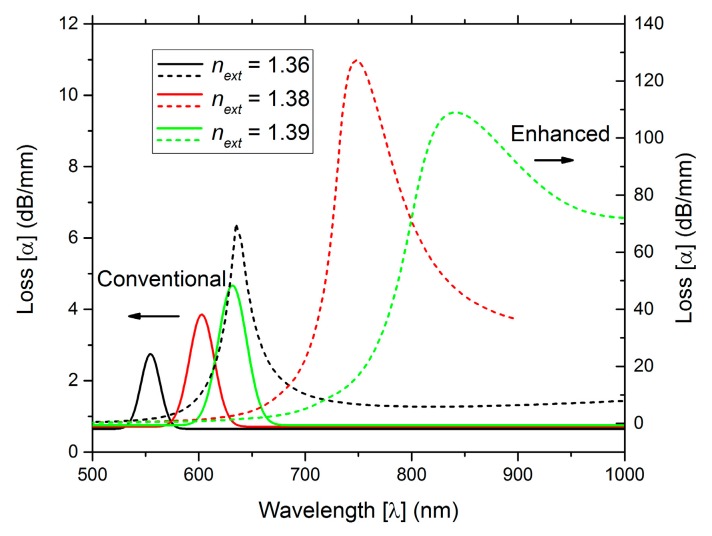
Loss vs wavelength for three external refractive indices.

Figure 2 represents the loss as function of wavelength for both configurations (conventional and PCF D-type fibers) for external refractive indices of *n_ext_* = {1.36, 1.38, 1.39}.

To determine the optical performance of the fiber sensors, the attenuation (loss) and the distribution of the Poynting vector in each fiber are observed. The configurations under comparison aim to increase the light interaction with the external medium (permitting more light to escape in this direction). From Figure 2 losses are higher for the PCF D-type fiber configuration, indicating a stronger plasmonic effect due to the higher energy transfer.

**Figure 3:**
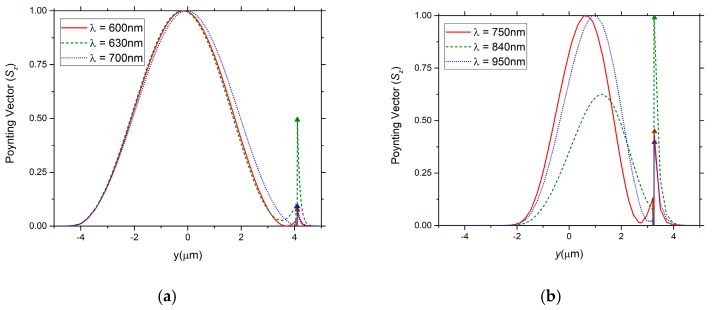
Normalized Poynting vector amplitude 1D across the fiber core: (**a**) for conventional D-type fiber structure and (**b**) for PCF D-type fiber structure (*n_ext_* = 1.39).

Figure 3 illustrates the normalized Poynting vector amplitude 1D across the fiber core, for an external refractive index *n_ext_* = 1.39 and for different wavelengths, considering (a) the conventional and (b) the PCF D-type fiber structures. The results indicate the PCF D-type fiber configuration allows a higher interaction of the light with the external medium, around a two times fold increase in the intensity of the Poynting vector.

For the ranges of *n_ext_* of [1.36, 1.38] and [1.38, 1.39], Table 1 compares both sensing configurations. For the conventional D-type fiber configuration, one metallic layer thickness is considered, *d_m_* = 45 nm, while for the PCF D-type fiber configuration two metallic layer thicknesses are considered, *d_m_* = 45 nm and *d_m_* = 65 nm, respectively. The resolution (R) and the sensitivity (S) were obtained considering an experimental spectral variation detection of 0.1 nm and using the data of Figure 2 and the following equations:

(22) $$S\left(\lambda \right)=\frac{\Delta \lambda_{peak}}{\Delta n_{ext}}$$

(23) $$R\left(\lambda \right)=\frac{1}{S\left(\lambda \right)}\Delta \lambda_{min}=\frac{\Delta n_{ext}\Delta \lambda_{min}}{\Delta \lambda_{peak}}$$

where *λ_min_* is the minimum wavelength value between two spectral lines that can be detected experimentally and *λ_peak_* is the wavelength shift of the resonance peak obtained from the simulations for different external refractive indices values (*n_ext_*).

The PCF D-type fiber sensor, with *d_m_* = 45 nm, presents a better performance, enhancing the sensitivity from 2.8 × 10^3^ to 10.2 × 10^3^ nm/RIU and the resolution from 3.6 × 10^−5^ to 9.8 × 10^−6^ RIU. The relevance of engineering the structure is confirmed by the results. The optimization of the available light energy near the metallic film, enhances the plasmonic effect, which in turn improves the sensitivity and resolution of the SPR sensor.

### 4.2. D-Type Fiber Configuration with Metallic Nanowire

Leaving the more conventional plasmonic sensing configuration that integrates a metallic film, addressed in the previous section, which supports plane wave plasmon modes, in this work we draw our attention to the insertion of a metallic nanowire that allows a sensor with strong localized plasmonic modes.

The existence of strong localized optical plasmonic modes introduce strong peaks in the loss spectrum and is a relevant element of the fiber sensor. These localized optical plasmonic modes are strongly guided and this light can be taken from the sensing section of the sensor, which results in a good coupling with conventional optical fibers. When the fiber sensor is integrated in a remote sensing system or in a sensing network this is of special importance. In order to emphasize the role of the plasmonic modes, it is assumed that guided modes result from conventional step index cores. 

**Figure 4:**
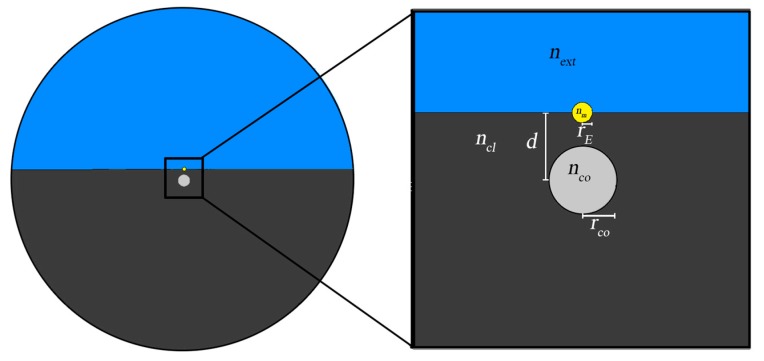
D-type fiber configuration with metallic nanowire, with a close-up inset of the relevant features.

Figure 4 [42] illustrates a novel design of a plasmonic D-type fiber sensor that integrates a gold nanowire mounted on the flat surface, substituting the conventional metallic film. As referred, the optical fiber has a conventional step index core (*n_co_*), made of silica doped with germanium (16%) and enclosed by a cladding of pure silica (*n_cl_*). The Drude model was used to calculate the gold effective refractive index (*n_m_*). In order to improve optical coupling to both the external medium (*n_ext_*) and to the core, as well as to introduce mechanical resistance to the plasmonic sensing configuration, the metallic nanowire was partially embedded in the fiber surface. The gold wire radius was *r_m_* = 300 nm, the core radius *r_co_* = 1 μm and the distance between the fiber core axis and the metal axis was *d* = 2 μm.

**Figure 5:**
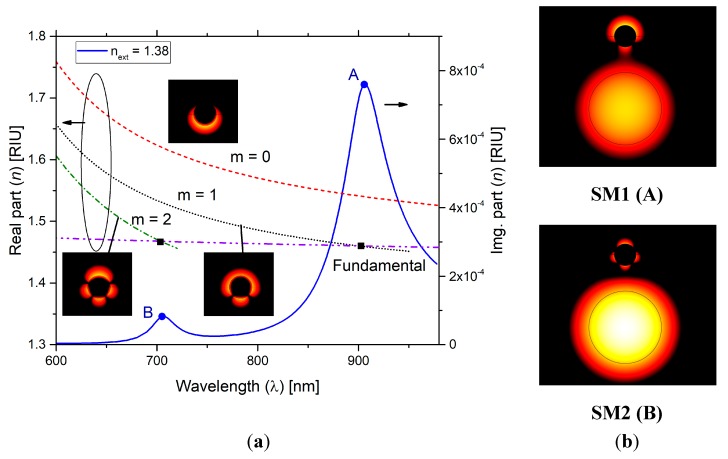
(**a**) Real and imaginary part of the refractive index as function of wavelength (*n_ext_* = 1.38). (**b**) Light intensity distribution for the supermodes SM1 and SM2.

The graph in Figure 5a illustrates the real part of the refractive index for the fundamental mode of the optical fiber and for the plasmon modes of the wire (*m* = 0, 1, 2). The intersection of these dispersion curves are indicated by squared dots and correspond, in wavelength, to the peaks of the curve of the imaginary part of the effective refractive index (solid blue line). These peaks result from a resonate coupling and are denoted as a supermode SM1, circle dot “A” and supermode SM2, circle dot “B”. Distribution of the intensity of the light in the metal wire for the plasmon modes of the wire are the detailed in the inset of Figure 5a. As referred, SM1 results from a high plasmonic interaction, noticeable at 900 nm, where the losses reach a high value (point A of Figure 5a) where the supermode presents a fairly strong dipole (upper image of Figure 5b).

After hybridization with the fiber core mode, the light intensity distribution for the supermodes SM1 and SM2 are shown in Figure 5b [43], revealing the transition between the two supermodes. A solid dipolar moment mode (peak A) and a solid quadrupole moment mode (peak B) are observable. Because of the higher losses and potentially higher sensitivity to external refractive index we turn our attention to peak A (supermode SM1).

We have carried out a comparison on the performance of this sensor with a conventional D-type fiber based design, also with *d* = 2 μm and *r_co_* = 1 μm, but with a metal film with 45 nm of thickness, mounted on the flat surface [1,44], in order to assess the advantages of this metallic nanowire configuration.

**Figure 6:**
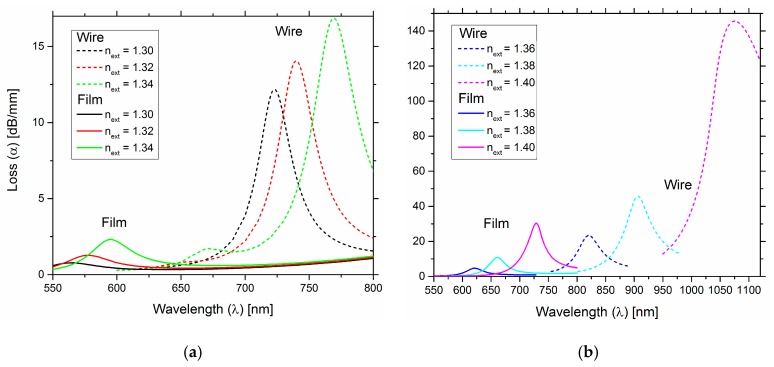
Loss curves versus wavelength comparison for two configurations, one for the metal nanowire and another for the more conventional configuration with a metal film, for two ranges of *n_ext_* = [1.30–1.34] (**a**) and *n_ext_* = [1.36–1.40] (**b**), respectively.

Therefore, the comparison results are presented in Figure 6a. They show that the metal nanowire supports two resonant peaks, that can be strongly wavelength shifted due to the alteration of the external refractive index under measurement, while the conventional configuration with a metal layer (film) it supports only a single resonant peak.

In Figure 6a, for the nanowire structure and considering the peak in the right—supermode SM1, for higher external refractive indexes, a fairly large wavelength shift is noticed, as well as an increase in the losses. The nanowire design structure in both graphs (Figure 6a,b) demonstrates higher losses, indicating a stronger coupling between the external medium and the plasmon and guided modes, than the conventional design structure with a metallic film.

Table 2 presents the results of sensitivity and resolution for various for ranges of *n_ext_*, for the two configurations under comparison.

The metallic nanowire design structure presented higher performance in comparison with the conventional metallic film design structure, both in sensitivity and resolution, for all ranges of *n_ext_* tested. As an example of performance improvement of using the plasmonic metallic nanowire structure, for *n_ex_*_t_ = [1.38, 1.40], the metallic nanowire sensor has a sensitivity of 8437 nm/RIU, while the conventional metal film sensor has a sensitivity of 3200 nm/RIU.

### 4.3. D-Type Fiber Configuration with Metamaterial

Localized modes can be found in other materials beyond metallic nanowires. For instance, some forms of metamaterials are made of compactly packed metal-dielectric structures with dimensions lower than the wavelength [13]. In this situation the aggregation of localized modes is so solid that they hybridized collectively, whose modes spread over the whole material. Consequently, the metamaterial acts as a bulk material, exhibiting characteristics that result from the nanoscale structure, and therefore can be tailored to stimulate the coupling with guided modes. Truly, the relevant feature of metamaterials is the capacity of exhibiting unusual optical characteristics that are not encountered in natural materials. For instance, metamaterials may present refractive indexes that stimulate the required boundary conditions to create surface plasmons at determined wavelengths or having optical properties that depend on the presence of certain environmental chemical species [16].

For the simulation modelling, the easiest strategy is to compute the optical properties of an ad-hoc metamaterial, using either numerical models [14], direct experimental measurement results [16] or analytical effective medium theory based models [13]. This permits to place the metamaterial as a bulk material in the numerical model. After that one can find the optimal parameters for the metamaterial that optimize sensor performance, such as size and shape, their relative position and composition of the nanostructures.

**Figure 7:**
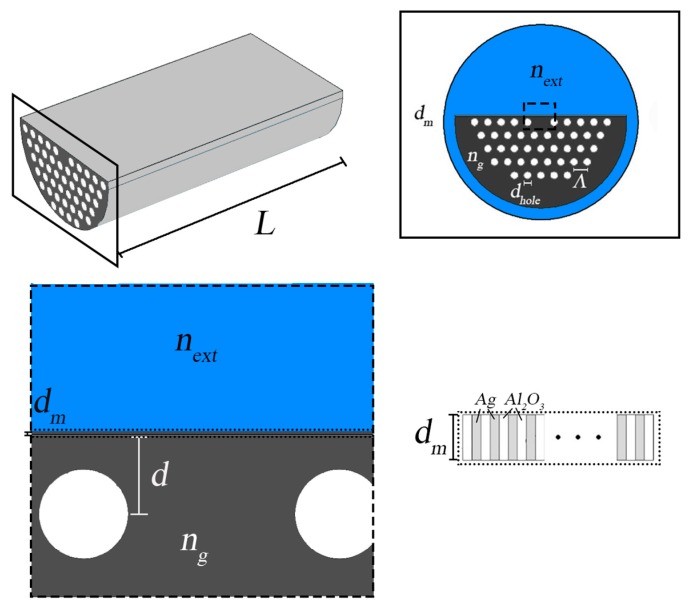
From upper left to lower right: 3D image of the fiber sensor, cross-section of the fiber, structure of the metamaterial surface and a scheme of the thin dielectric-metal layers that constitute the metamaterial.

In this work, it is considered a D-type PCF refractive index sensor based on SPR with a planar structure, where the conventional metal film is replaced by a metamaterial. The performance analysis and a comparison with the conventional structure is conducted. The metamaterial film is composed of thin layers of alumina and silver, as shown in Figure 7 [45].

**Figure 8:**
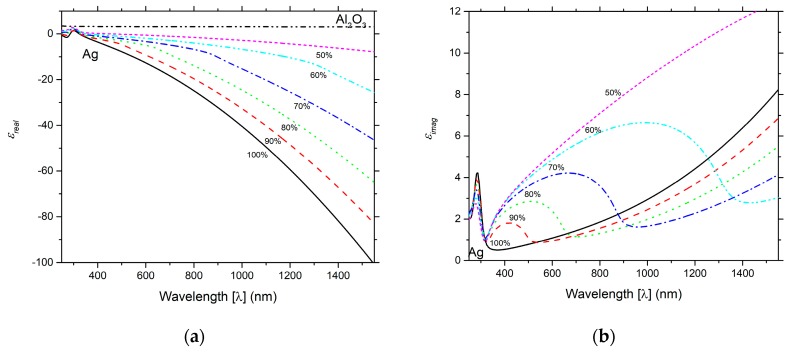
Complex dielectric (**a**) real part and (**b**) imaginary part of the metamaterial for concentrations of: 50% (small dashed curve), 60% (dash-double dotted curve), 70% (dash-dotted curve), 80% (dotted curve), 90% (dashed curve), and Ag of 100% (solid curve, corresponding to pure silver).

The relative thickness of the alumina and silver layers define the metamaterial optical properties. Using an effective medium approach, it is possible to calculate the effective refractive index of the metamaterial. For that, the most commonly used models are the Maxwell-Garnett Theory and the Bruggeman effective medium theory [46]. Figure 8 illustrates the real (Figure 8a) and imaginary (Figure 8b) parts of the dielectric, for several filling ratios concentrations of Al_2_O_3_ and Ag, calculated using the Bruggeman effective medium theory model. The metamaterial films with fill ratios from 100% (the case of a pure silver film) until 50%, display a negative dielectric constant for the vast majority of the spectral range considered. Such condition is necessary to promote the existence of surface plasmons. For a fill ration of 50%, the formed metamaterial approaches the epsilon-near-zero regime, limiting the validation of the effective medium approach.

**Figure 9:**
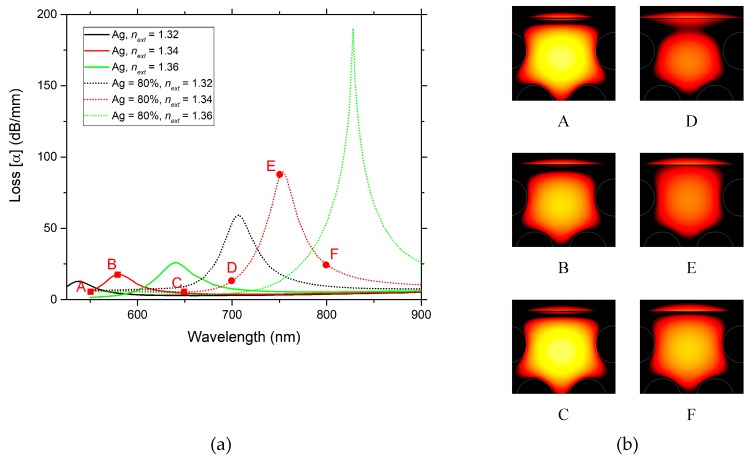
(**a**) Loss as function of wavelength for two different layers, one with only Ag and the other with the metamaterial considered (80% Ag and 20% Al_2_O_3_), when subjected to different *n_ext_* values. (**b**) Light distribution in the core fiber region and in the region of the metal/metamaterial film for *n_ex_*_t_ = 1.34 and for two concentrations of Ag (100% and 80%). The pure silver film is in the left column and the metamaterial film is in the right column.

In Figure 9, we compare results of the loss as function of wavelength for the metamaterial (80% of Ag and 20% of Al_2_O_3_) and for pure silver (only Ag), when subject to different external refractive indices. The peaks of loss, distinctive characteristic of SPR are intensely affected by the external refractive index when the metamaterial layer is considered, while for the case of pure silver they are not vividly affected by the external refractive index change.

From the light distribution present in Figure 9b, it is possible to state that light is less concentrated in the core for the metamaterial film, which permits the formation of a stronger plasmon, promising in a clear way better results when the silver film is substituted by the metamaterial film. Table 3 presents, for different external refractive indices, respectively, the sensor sensitivity and sensor resolution for pure silver and for the metamaterial with a concentration of 80%Ag and 20%Al_2_O_3_.

The results show clearly the possibility of performance improvement, in sensitivity and resolution, of SPR optical fiber sensors when using metamaterials. The metamaterial is able to act as an artificial metal, whose optical properties are not found in natural materials. Moreover, the metamaterial can be tailored to optimize the sensor performance characteristics.

### 4.4. D-Type Fiber Configuration with Internal and External Metallic Nanowires for Simultaneous Measurements 

Evidently, the guided mode can couple to more than just a single localized plasmonic mode. Therefore, a plasmonic fiber sensor can integrate structures whose respective localized plasmonic modes are sensitive to distinct analytes or parameters, or the distinct analytes or parameters can couple with the guided mode at distinct wavelengths, permitting the development of sensors capable of detecting multiparameters. Due to the intrinsic nature of the plasmonic resonant coupling mechanism, it is important to assure the coupling between the localized plasmonic modes and the guided mode occurs at a suitably wavelength separation, in order to minimize any possible cross-talk and guarantee independent detection.

However, it is important to state that it is possible to couple the guided mode and different localized plasmon modes at the same wavelength (a form of degenerated mode coupling or hybridization) to enhance the sensing mechanism. In this situation, the individual plasmon modes are hybridized and form cooperative plasmon modes with different optical properties that couple with guided mode. This cooperative plasmon modes can reinforce their dependence on the external parameters and augment the sensor performance. One possible example is the configuration shown in sub-section “D-type fiber configuration with metallic nanowire” where the single nanowire would be accompanied by several nanowires, placed adjacently to the original nanowire.

**Figure 10:**
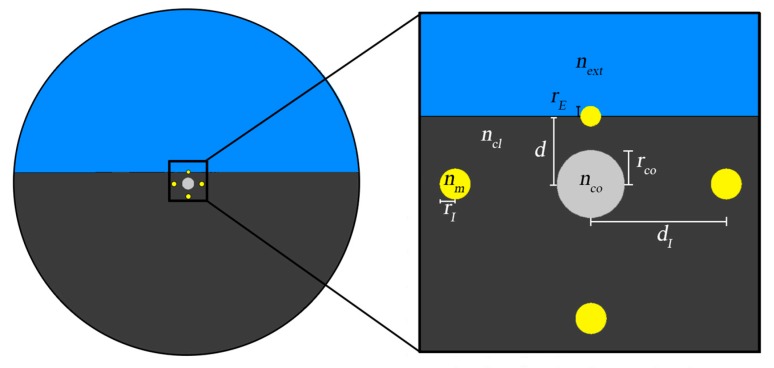
Configuration of the proposed SPR D-type fiber with one external and three internal metallic nanowires for simultaneous measurement.

In this sub-section it is analyzed a sensing configuration composed by an engineered D-type fiber with a set of metallic nanowires, where the generated surface plasmon resonances allow to measure refractive index and temperature simultaneously [47]. Figure 10 presents the configuration of the sensor which is based on a D-type fiber with three gold nanowires mounted in the fiber cladding, around the core and one gold nanowire implanted on the flat surface. This latter nanowire is partially embedded on the fiber cladding flat surface to enhance mechanical resistance and to allow optical coupling to the external medium, and therefore exposed to the external refractive index. The three inner metal nanowires are not exposed to the external refractive index and therefore their localized plasmon modes will be sensitive only to temperature.

The important geometrical parameters are the following: distance between the external metallic nanowire center and the fiber core center (*d* = 2 μm), the core radius (*r_co_* = 1 μm), the internal metallic nanowires radii (*r_I_* = 500 nm) and the metallic external nanowire radius (*r_E_* = 300 nm).

**Figure 11:**
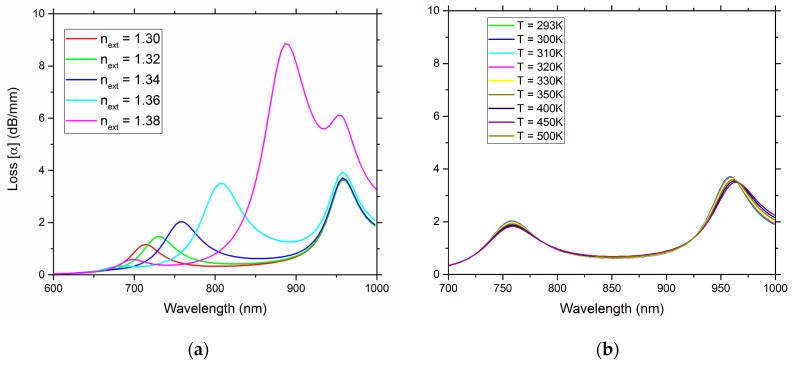
Loss versus wavelength of the sensor for: different external refractive index values, for a constant temperature of 293 K (**a**); different temperature values, with a constant external refractive index of 1.34 (**b**).

Figure 11a illustrates the guided mode loss, per unit of length, versus wavelength for distinct external refractive index values, revealing three main peaks. The first peak at low wavelengths is extremely weak (λP_1_, present at 700 nm with *n_ext_* = 1.38) and it is not useful for sensing. The higher peak is the second one (λP_2_, present at 900 nm, with *n_ext_* = 1.38) and shifts towards longer wavelengths when the external refractive index increases, and finally the third peak (λP_3_, present at 950 nm for *n_ext_* = 1.38) remains practically unchanged when the external refractive index changes. Therefore, this third peak is the adequate peak for temperature measurements. This third peak is weaker in magnitude than the second one, a property that can be tailored either by altering the number of internal nanowires, or by changing the distance between the core and the internal nanowires. Another option is to lower the second peak intensity by increasing the distance between the external wire and the core.

Figure 11b illustrates the loss versus wavelength for different temperature values. The left peak corresponds to the second peak (λP_2_) present in Figure 11a, while the right peak corresponds to the third peak (λP_3_) present in Figure 11a. The wavelength of λP_2_ changes with the external refractive index and remains practically unchanged with the temperature. On the contrary, λP_3_ does not depend on the external refractive index, and for higher temperatures shows a shift toward longer wavelengths. 

In Table 4 it is presented the sensitivity coefficients of the for refractive index and temperature wavelength peaks, clearly indicating the capacity of this D-type fiber sensor configuration, with gold nanowires, to simultaneously measure refractive index and temperature, based on localized plasmons.

This work has proved the possibility to engineer the fiber structure with metal wires to generate localized plasmon modes in the wires, that result in two distinct measurement peaks, capable of simultaneously measuring two distinct physical parameters. In principle, this concept can be further extended to measure even more parameters.

## 5. Future Perspectives

The continuous development of fabrication techniques allowed to conceive a new generation of nanostructured optical fiber sensors, that explore a multitude of new degrees of freedom and control of the operation of these devices. The size, shape and material constitution fix the optical characteristics of the localized modes supported by the metal-dielectric structures, while their relative position controls how they couple and hybridize to determine the optical response of the sensor to external parameters or analytes. In this article, we have shown a few examples of how to explore this tremendous potential using numerical tools, but much of the possibilities and combinations have only now begun to be explored and much work is still to be performed.

It has been shown how photonic crystal fibers can be used to generate well localized and guided optical modes that carry light into the sensor and provide a readout channel. In general, for sensors based on the measurement of losses, we are looking for guided modes with very low losses and therefore optical confinement is of crucial importance. Then, at specific wavelengths it is possible to introduce loss peaks, via the resonant coupling with other modes, either localized plasmonic modes or collective modes that are responsive to the parameters being monitored. The challenge here is to engineer these modes to simultaneously create a strong dependency on the external parameters while still satisfying the necessary conditions for a resonant coupling with the localized guided modes. Clearly, the range of structural parameters of the sensor is so wide and the costs of fabrication so large that producing and testing each design is unpractical, leaving numerical solutions as the main tool for sensor performance optimization. 

In this review paper, the focus was on a specific subset of nanostructured fiber optical sensors, namely those that have a metal-dielectric nanostructured cross-section uniform throughout the length of the sensing section of the optical fiber and that operate via the monitoring of the optical losses. In these cases, the problem can be reduced to the analysis of a two-dimensional eigen-value problem associated with the modes supported by the device, and ultimately in the determination of the imaginary part of the effective refractive index for each mode. Clearly, the FEM constitutes the ideal method to solve numerically this problem by balancing accuracy with economy of computer resources, specifically computer memory and run time. This approach can be extended to nanostructures much more complex than those presented here, but they constitute examples of the phenomena at hand and how to employ them in sensor design using numerical tools. Specifically, it was exemplified the use of nanowires, metamaterials and photonic crystal fibers, but much more is possible. 

These basic principles described in this paper can be adapted to consider more complex situations. One can conceive three dimensional structures that introduce non-uniform distributions of refractive index throughout the length of the optical fiber. These problems will be computationally much more intensive, especially if these structures do not have a repetitive pattern that allow to divide the fiber into cell structures and employ FEM with periodic boundary conditions, much alike the treatment given to study of crystals. Then, it will be necessary to combine beam propagation methods to fully account for the evolution of the optical signal as it propagates through the fiber.

On the other hand, the micro and nanoscale of these structures forces the discretization of the optical modes supported and in some sense these modes become quantized, of which the similarity with the hybridization of atomic orbitals during the formation of a chemical bond constitutes an intuitive clue. In this case, the system is not truly quantum but behaves much like a quantum system. This similarity suggests that it might be possible to combine these structures with truly quantum effects, such mode squeezing, to enhance sensing sensitivity. This would require a completely new approach to the experimental methods used to operate the sensor and necessarily in the modeling tools employed. 

Also, it is important to notice that plasmons are inherently nonlinear excitations and as we consider structures with sizes and separations of just a few tens of nanometers these effects might be noticeable, even at low optical powers. Capturing these effects and employing them in optical sensing will require yet a new challenge into numerical modeling. To provide an example, if instead of using the refractive index to model the properties of the metal, we use a fluid description for the motion of the free or conduction electron in the material then, we would obtain necessarily a system of coupled differential equations that are inherently nonlinear.

In all these situations, whether we consider simpler or more complex nanostructured optical sensors, or even quantum and nonlinear effects, the exploration in design and engineering of these sensors requires numerical simulation tools of ever-increasing sophistication. It constitutes a challenge for both those who advance new sensors and to those who develop these simulation tools. 

## Figures and Tables

**Table 1:** Sensitivity and resolution values for both configurations.

	Sensitivity (nm/RIU)			Resolution (RIU)		
Range of *n_ext_*	Conventional	PCF -45 nm	PCF -65 nm	Conventional	PCF -45 nm	PCF -65 nm
[1.36,1.38]	2.40 × 10^3^	5.20 × 10^3^	5.30 × 10^3^	4.20 × 10^−5^	1.90 × 10^−5^	1.90 × 10^−4^
[1.38,1.39]	2.80 × 10^3^	10.2 × 10^3^	8.20 × 10^3^	3.60 × 10^−5^	9.80 × 10^−6^	1.20 × 10^−4^

**Table 2:** Comparison of the sensitivity and resolution for the two structures, one with metallic film and the other one with the metallic wire.

	Sensitivity (nm/RIU)		Resolution (RIU)	
Range of *n_ext_*	Film	Wire	Film	Wire
[1.30,1.32]	700	891	1.4 × 10^−5^	1.1 × 10^−5^
[1.32,1.34]	900	1437	1.1 × 10^−5^	7.0 × 10^−6^
[1.34,1.36]	1300	2562	7.7 × 10^−6^	3.9 × 10^−6^
[1.36,1.38]	2000	4287	5.0 × 10^−6^	2.3 × 10^−6^
[1.38,1.40]	3200	8437	3.1 × 10^−6^	1.2 × 10^−6^

**Table 3:** Sensor sensitivity and sensor resolution for the D-type SPR refractive index optical fiber sensor with two different films, silver and the metamaterial with 80%Ag and 20%Al_2_O_3_, respectively.

	Sensitivity (nm/RIU)		Resolution (RIU)	
Range of *n_ext_*	Ag	Metamaterial	Ag	Metamaterial
[1.30,1.32]	1.75 × 10^3^	2.30 × 10^3^	5.88 × 10^−5^	4.35 × 10^−5^
[1.32,1.34]	2.00 × 10^3^	2.40 × 10^3^	4.76 × 10^−5^	4.17 × 10^−5^
[1.34,1.36]	2.80 × 10^3^	3.70 × 10^3^	3.57 × 10^−5^	2.70 × 10^−5^

**Table 4:** Sensitivity coefficients for the refractive index and temperature peaks.

	K_n_ (nm/RIU)	K_T_ (pm/K)
$\Delta \lambda_{P2}$	2093.1	3.4
$\Delta \lambda_{P3}$	0	27.3

## References

[1] Lee B., Roh S., Park J. (2009). Current status of micro- and nano-structured optical fiber sensors. Opt. Fiber Technol..

[2] Monzón-Hernández D., Villatoro J. (2006). High-resolution refractive index sensing by means of a multiple-peak surface plasmon resonance optical fiber sensor. Sens. Actuators B Chem..

[3] Moayyed H., Leite I.T., Coelho L., Santos J.L., Guerreiro A., Viegas D. Analysis of phase interrogation of SPR fiber optic sensors with characteristics tailored by the application of different metal-dielectric overlays. Proceedings of the 23rd International Conference on Optical Fiber Sensors.

[4] Moayyed H., Leite I.T., Coelho L., Santos J.L., Viegas D. (2014). Analysis of Phase Interrogated SPR Fiber Optic Sensors with Bimetallic Layers. IEEE Sens. J..

[5] Čtyroký J., Homola J., Skalský M. (1997). Modelling of surface plasmon resonance waveguide sensor by complex mode expansion and propagation method. Opt. Quantum Electron..

[6] Lee B., Roh S., Kim H., Jung J., Yin S., Guo R. (2009). Waveguide-based surface plasmon resonance sensor design. Photonic Fiber and Crystal Devices: Advances in Materials and Innovations in Device Applications III.

[7] Viegas D., Hautakorpi M., Guerreiro A., Santos J., Ludvigsen H. Surface-Plasmon-Resonance Sensor Based on H-shaped Optical Fiber Surface-Plasmon-Resonance Sensor Based on H-shaped Optical Fiber. Proceedings of the Fourth European Workshop on Optical Fiber Sensors.

[8] Kaliteevski M.A., Nikolaev V.V., Abram R.A. (2000). Calculation of the Mode Structure of Multilayer Optical Fibers Based on Transfer Matrices for Cylindrical Waves. Opt. Spectrosc..

[9] Hoa X.D., Tabrizian M., Kirk A.G. (2009). Rigorous Coupled-Wave Analysis of Surface Plasmon Enhancement from Patterned Immobilization on Nanogratings. J. Sens..

[10] Nogueira Coelho T.V.N., Carvalho J.P., Pontes M.J., Santos J.L., Guerreiro A. Remote optical fiber sensor based on an LPG sensor head with Raman amplification optimized by numerical methods. Proceedings of the Optical Sensing and Detection II.

[11] Pontes M.J., Coelho T.V.N., Carvalho J.P., Santos J.L., Guerreiro A., Martins Costa M.F.P.C. (2013). Remote fiber sensors and optical amplification. Proceedings of the 8th Iberoamerican Optics Meeting and 11th Latin American Meeting on Optics, Lasers, and Applications.

[12] Kuo S.M., Huang Y.W., Yeh S.M., Cheng W.H., Lin C.H. Liquid crystal modified photonic crystal fiber (LC-PCF) fabricated with an SU-8 photoresist sealing technique for electrical flux measurement. Proceedings of the IEEE International Conference on Micro Electro Mechanical Systems (MEMS).

[13] Silva A.O., Leite I.T., Teixeira J.M., Araujo J.P., Costa J.C.W.A., Giraldi M.T.R., Jorge P.A.S., Guerreiro A. Effective medium theory of subwavelength arrays of metallic nanowires: A numerical approach based on modal propagation method. Proceedings of the 8th Iberoamerican Optics Meeting and 11th Latin American Meeting on Optics, Lasers, and Applications.

[14] Fernandes P., Leite I.T., Teixeira J.M., Hierro-Rodríguez A., Araújo J.P., Guerreiro A. Sensing with metallic nanowires?. Proceedings of the 23rd International Conference on Optical Fiber Sensors.

[15] Leite I.T., Fernandes P., Hierro-rodríguez A., Teixeira J.M., Jorge P.A.S., Guerreiro A. Analysis of a fiber-optic sensor design based on SPR in nanowire metamaterial films. Proceedings of the 23rd International Conference on Optical Fibre Sensors.

[16] Hierro-Rodriguez A., Leite I.T., Rocha-Rodrigues P., Fernandes P., Araujo J.P., Jorge P.A.S., Santos J.L., Teixeira J.M., Guerreiro A. (2016). Hydrogen sensing via anomalous optical absorption of palladium-based metamaterials. Nanotechnology.

[17] Al-Qazwini Y., Arasu P.T., Noor A.S.M. Numerical investigation of the performance of an SPR-based optical fiber sensor in an aqueous environment using finite-difference time domain. Proceedings of the 2011 2nd International Conference on Photonics.

[18] Hautakorpi M., Mattinen M., Ludvigsen H. Surface-plasmon-resonance sensor based on suspended-core microstructured optical fiber. Proceedings of the OECC/ACOFT 2008—Joint Conference of the Opto-Electronics and Communications Conference and the Australian Conference on Optical Fiber Technology.

[19] Santos D.F., Guerreiro A., Baptista J.M. (2013). Numerical investigation of a refractive index SPR D-type optical fiber sensor using COMSOL multiphysics. Photonic Sens..

[20] Santos D.F., Guerreiro A., Baptista J.M. Performance analysis simulation of new SPR microstructured D-type optical fiber sensor configurations for refractive index measurement. Proceedings of the 23rd International Conference on Optical Fiber Sensors.

[21] Tyagi H.K., Lee H.W., Uebel P., Schmidt M.A., Joly N., Scharrer M., Russell P.S.J. (2010). Plasmon resonances on gold nanowires directly drawn in a step-index fiber. Opt. Lett..

[22] Uebel P., Schmidt M.A., Scharrer M., Russell P.S.J. (2011). An azimuthally polarizing photonic crystal fiber with a central gold nanowire. New J. Phys..

[23] Kim H., Lee I.-M., Lee B. (2007). Extended scattering-matrix method for efficient full parallel implementation of rigorous coupled-wave analysis. J. Opt. Soc. Am. A.

[24] Špačková B., Piliarik M., Kvasnička P., Themistos C., Rajarajan M., Homola J. (2009). Novel concept of multi-channel fiber optic surface plasmon resonance sensor. Sens. Actuators B Chem..

[25] Sharma A.K., Gupta B.D. (2007). On the performance of different bimetallic combinations in surface plasmon resonance based fiber optic sensors. J. Appl. Phys..

[26] Sharma A.K., Jha R., Gupta B.D. (2007). Fiber-Optic Sensors Based on Surface Plasmon Resonance: A Comprehensive Review. IEEE Sens. J..

[27] Bhatia P., Gupta B.D. (2011). Surface-plasmon-resonance-based fiber-optic refractive index sensor: Sensitivity enhancement. Appl. Opt..

[28] Moayyed H., Leite I.T., Coelho L., Santos J.L., Viegas D. (2015). Theoretical Study of Phase-Interrogated Surface Plasmon Resonance Based on Optical Fiber Sensors with Metallic and Oxide Layers. Plasmonics.

[29] Perrotton C., Javahiraly N., Slaman M., Dam B., Meyrueis P. (2011). Fiber optic Surface Plasmon Resonance sensor based on wavelength modulation for hydrogen sensing. Opt. Express.

[30] Lesina A.C., Vaccari A., Berini P., Ramunno L. (2015). On the convergence and accuracy of the FDTD method for nanoplasmonics. Opt. Express.

[31] Kaye S., Zeng Z., Sanders M., Chittur K., Koelle P.M., Lindquist R., Manne U., Lin Y., Wei J. (2017). Label-free detection of DNA hybridization with a compact LSPR-based fiber-optic sensor. Analyst.

[32] Axelevitch A., Apter B., Golan G. (2013). Simulation and experimental investigation of optical transparency in gold island films. Opt. Express.

[33] Pomplun J., Zschiedrich L., Klose R., Schmidt F., Burger S. (2007). Finite element simulation of radiation losses in photonic crystal fibers. Phys. Status Solidi.

[34] Popescu V.A., Puscas N.N., Perrone G. (2017). Simulation of the Sensing Performance of a Plasmonic Biosensor Based on Birefringent Solid-Core Microstructured Optical Fiber. Plasmonics.

[35] Luan N., Wang R., Lv W., Yao J. (2015). Surface plasmon resonance sensor based on D-shaped microstructured optical fiber with hollow core. Opt. Express.

[36] Peng L., Shi F., Zhou G., Ge S., Hou Z., Xia C. (2015). A Surface Plasmon Biosensor Based on a D-Shaped Microstructured Optical Fiber with Rectangular Lattice. IEEE Photonics J..

[37] Tan Z., Li X., Chen Y., Fan P. (2014). Improving the Sensitivity of Fiber Surface Plasmon Resonance Sensor by Filling Liquid in a Hollow Core Photonic Crystal Fiber. Plasmonics.

[38] Erdmanis M., Viegas D., Hautakorpi M., Novotny S., Santos J.L., Ludvigsen H. (2011). Comprehensive numerical analysis of a surface-plasmon-resonance sensor based on an H-shaped optical fiber. Opt. Express.

[39] Hassani A., Skorobogatiy M. (2006). Design of the microstructured optical fiber-based surface plasmon resonance sensors with enhanced microfluidics. Opt. Express.

[40] Yao J., Yang X., Wang M., Lu Y. (2015). Surface plasmon resonance sensor based on hollow-core PCFs filled with silver nanowires. Electron. Lett..

[41] Santos D.F., Guerreiro A., Baptista J.M. (2015). SPR Microstructured D-Type Optical Fiber Sensor Configuration for Refractive Index Measurement. IEEE Sens. J..

[42] Santos D.F., Guerreiro A., Baptista J.M. (2017). Surface plasmon resonance sensor based on D-type fiber with a gold wire. Optik.

[43] Schmidt M.A., Russell P.S. (2008). Long-range spiralling surface plasmon modes on metallic nanowires. Opt. Express.

[44] Dostálek J., Čtyroký J., Homola J., Brynda E., Skalský M., Nekvindová P., Špirková J., Škvor J., Schröfel J. (2001). Surface plasmon resonance biosensor based on integrated optical waveguide. Sens. Actuators B Chem..

[45] Santos D.F., Guerreiro A., Baptista J.M. (2017). SPR optimization using metamaterials in a D-type PCF refractive index sensor. Opt. Fiber Technol..

[46] Cai W., Shalaev V. (2010). Optical Metamaterials.

[47] Santos D.F., Guerreiro A., Baptista J.M. (2017). Simultaneous Plasmonic Measurement of Refractive Index and Temperature Based on a D-Type Fiber Sensor with Gold Wires. IEEE Sens. J..

